# Medicinal Herbals with Antiplatelet Properties Benefit in Coronary Atherothrombotic Diseases

**DOI:** 10.1155/2016/5952910

**Published:** 2016-03-14

**Authors:** Nurul Huda Mohd Nor, Fauziah Othman, Eusni Rahayu Mohd Tohit, Sabariah Md Noor

**Affiliations:** ^1^Department of Human Anatomy, Faculty of Medicine and Health Sciences, Universiti Putra Malaysia, 43000 Serdang, Selangor, Malaysia; ^2^Department of Pathology, Faculty of Medicine and Health Sciences, Universiti Putra Malaysia, 43400 Serdang, Selangor, Malaysia

## Abstract

Coronary atherothrombotic diseases such as coronary artery disease, peripheral vascular disease, cerebrovascular disease, and heart failure are the serious concerns of the thrombus formed in blood vessels. Anticoagulant and antiplatelet drugs are the cornerstones of the management of these diseases. To prevent the recurrence of these diseases, double antiplatelet drugs such as aspirin and clopidogrel has been the standard management in most hospitals. However, aspirin resistance and clopidogrel inefficient effects due to noncompliance with double drugs regimen can cause a sinister effect on patients. Medicinal plants serve as a greater resource for new medication and their potential currently became a topic of interest to the researchers all over the world. Traditionally, certain herbs have been used as a treatment for heart diseases but have been investigated for their antiplatelet properties. This current review explained few traditional antithrombotic herbals and their antiplatelet properties* in vitro* and* in vivo *and this is to be deeply discussed in further research.

## 1. Introduction

Coronary atherothrombotic diseases (CADs) are the leading causes of death and disabilities in the world. Globally, it is estimated that about 17.3 million people had died from CADs in 2008 and, by 2030, approximately 23.6 million of the world population might die from CADs [1]. CADs consist of coronary artery disease, peripheral vascular disease, cerebrovascular disease, and heart failure. The risk factor of CADs is mainly due to atherosclerosis that leads to arterial thrombosis. The pathogenesis process encompasses chronic progressive atherosclerosis punctuated by acute processes, that is, (1) plaque rupture and (2) platelet thrombus formation in the areas of progressive stenosis. The final pathway to arterial thrombosis is atherosclerosis plaque rupture; thus, the effective way to treat or to prevent this occurrence is by limiting or eradicating platelet-dependent thrombus formation.

Oral antiplatelet drugs are the cornerstone of the therapy of CADs that consist of primary and secondary prevention strategies to manage these diseases. Patients with efficient antiplatelet therapy can reduce stroke and death rates by about 25% [2]. Currently, the standard practice in a clinical setting to prevent adverse events of CADs is a combination of aspirin and clopidogrel which inhibits cyclooxygenase and antagonizes P2Y_12_, respectively. Despite their proven effectiveness, there are cases of recurrent cardiovascular diseases among those who take double antiplatelet drugs. This might be due to incompliancy and the adverse effect faced by the patients. Gastrointestinal bleeding and gastric ulcers are the most common adverse effects reported [3]. There are three points of argument of aspirin resistance that include the following: no evidence of antithrombotic effect being aspirin dosage related, ability to generate thromboxane A2 despite the usual aspirin dosage, and low dose of aspirin causing blocking of more than 95% of platelet cyclooxygenase-1 activity. Thus, inability to benefit from regular aspirin dosage leads to the failure of aspirin as an antiplatelet therapy [4].

Medicinal plants serve as a greater resource for new medication and their potential currently became a topic of interest to the researchers all over the world. The World Health Organisation (WHO) has recommended medicinal plants to be used more effectively in healthcare system [1]. Recently, there is a trend of studies on the development of natural compound present in traditional medicinal herbs demonstrating antiplatelet properties. The currently known mechanisms of action that can be used to interfere with the primary haemostatic system that regulates receptors such as catecholamine, collagen, thrombin, prostacyclin, ADP, PGD_2_, PGE_2_, and serotonin, TXA_2_, cAMP, cGMP, and calcium have been identified to be the effective target of antiplatelet therapy [5]. In order to have a wider variety of treatments, screening of medicinal herbs that is postulated to have high potential of antiplatelet properties was performed.

## 2. Methods

The information about medicinal plants that are traditionally used in the management of coronary atherothrombotic disease was obtained from published papers and texts on ethnobotanical studies as well as those investigating the effects of plant(s) used with anticoagulation effect. A literature search on electronic databases such as Google Scholar, PubMed, and Scopus up to 2015 was carried out using “antiplatelet” and “herbal” as the keywords for primary search and then “plant name – accepted or synonyms”, “constituents”, and/or “mechanism” for the secondary search.

In order to highlight the medicinal plants traditionally used in CADs management with the potential of integration in the healthcare system, not all identified plants were included in this paper. Therefore, this review was not exhaustive of all plants used traditionally for treating CADs.

## 3. Results and Discussions

CAD can be prevented or treated by interfering with its mechanism of thrombosis. The primary goal of antithrombotic therapy is to inhibit platelet activation (primary haemostasis) and fibrin formation via cascade (secondary haemostasis) from happening. There are four steps in platelet activation pathways which are adhesion, aggregation, secretion, and procoagulation which lead to secondary haemostasis. However, this review focuses on primary haemostasis and the main method used in these various studies was light impedance aggregometry, either* in vivo* or* in vitro*. There are a lot of plants that exhibit antiplatelet properties. Ten plants from various families and regions are discussed as they possibly contain the compound responsible for those properties as listed in Table 1.

### 3.1. *Curcuma longa* (Turmeric)

Turmeric is derived from the rhizomes of* Curcuma longa* and has been used in Southeast Asia for a long time as an Indian spice and treatment for several conditions such as stomachache, wounds, and joint problems in Ayurvedic medicine [6]. Turmeric itself has been extensively used for its antioxidant, antimicrobial, anti-inflammatory, and antiproliferative properties. The most lipophilic component of* Curcuma longa* extracted called Curcuma oil (C. oil) has been utilized as antimicrobial, antifungal, antiviral, and anti-inflammatory agent and also used for wound healing activity.

A fraction of C. oil, namely, ar-turmerone, has been shown to inhibit collagen- (10 *µ*g/ml), adenosine-5′-diphosphate- (ADP- (10 *µ*M)), and thrombin- (0.64 U/ml) induced platelet aggregation. The effect of C. oil on platelet aggregation (*in vivo*) in rats significantly reduced collagen- (28%), ADP- (31%), and thrombin- (34%) induced platelet aggregation in comparison to aspirin (50 to 75%). While these three agonists had significantly inhibitory effect, calcium ionophore, PAF, and arachidonic acid were not affected at all [6]. This study is supported by another study done on ar-turmerone that showed that ar-turmerone inhibited platelet activation induced by collagen (2 *µ*g/ml) gave 100% inhibition at concentration of 100 *µ*g/ml and 80% and 60% inhibition at concentration of 10 *µ*g/ml and 5 *µ*g/ml, respectively [7]. In a study that used curcumin (500 *µ*M), a major ingredient of* Curcuma longa* showed effective inhibition against AA-induced platelet aggregation (1.0 mM). A further study has noted that an increase in lipoxygenase- (LOX-) derived products was observed in curcumin-treated platelets [8]. These could be due to the redirection of AA to LOX pathway or/and potentiation of the enzyme. AA is a membrane-derived fatty acid metabolized by cyclooxygenase to prostaglandin (PG) endoperoxide intermediates such as PGH_2_. In platelets, endoperoxides are further metabolized to thromboxane A_2_ and PGs [9]. This suggests that the antiplatelet effect of C. oil possibly affects the cyclooxygenase (COX) pathway in platelet at membrane/receptor level.

### 3.2. *Berberis vulgaris*

Scientifically named as* Berberis vulgaris*, the herb known as “barberry” is a native herb in Europe and Asia and is wildly grown in Canada to Pennsylvania. In traditional Chinese medicine, barberry was mentioned more than 3000 years ago to have diverse medicinal properties including antimicrobial, antiemetic, antipyretic, and antipruritic properties. Scientifically, barberry may offer healthcare benefits as an antihypertensive and vasodilator agent [10] and play a prominent role in apoptosis in the treatment of hepatocarcinogenic rats [11].

A study has been done to investigate the antiplatelet effects of* Berberis vulgaris* on alkaloid component named berberine (BR) towards rabbits as* in vivo* model. In the study, platelet aggregation induced by adenosine diphosphate (ADP), collagen, AA, and calcium ionophore A23187 was differentially inhibited in which collagen-induced platelet aggregation was most potently inhibited and calcium ionophore A23187-induced platelet aggregation was least inhibited. The inhibition of platelet aggregation is dose dependent from 10 to 30 mg/kg, giving the maximum inhibition of 52.5% [12]. There is also another report of inhibitory effects on AA metabolism and calcium influx, partial agonist activity on platelet *α*2-adrenoceptors using* Berberis vulgaris* extract [13].

### 3.3. *Osmanthus fragrans*

This plant has been used as an additive in beverage and also in perfumes as it has intense fragrance [31]. Besides, it had been used traditionally as treatment's constituent for stomachache, rheumatism, and halitosis [14].* Osmanthus fragrans* (OF) has high amount of phenolic acid which has been used as a natural source of antioxidant for controlling probing free radicals related to various diseases including chronic inflammation, cataracts, and atherosclerosis and other cardiovascular diseases [32].

One novel study on phytochemical investigation on OF seed, the purple-brown ripe fruit of OF, extracted using ethyl acetate and n-butyl alcohol showed the greatest effect on inhibition of collagen-induced platelet aggregation at concentration of 1.4 mg/ml and 2.8 mg/ml. Further study using the same extracts but using ADP showed equal effect compared to a positive drug at 2.8 mg/ml [14]. These findings suggest that OF seeds may have the potential to be developed as a novel inhibitor of platelet aggregation.

### 3.4. *Magnolia officinalis*

This traditional Chinese and Japanese medicinal herb comes from Magnoliaceae family and is being used for the treatment of thrombotic stroke, headache, and typhus fever [15]. It is believed to have multifunctional health benefits including general antioxidative effects through scavenging hydroxyl radicals that are generated by ultraviolet (UV) irradiation [33] and anticancer properties induced by apoptosis of HER2 overexpressed in breast cancer cells [34]. They are also capable of controlling pain [35]. Besides that, magnolol has an antiapoptotic effect through the regulation of bcl-XL gene and suppression of bcl-xS gene [36].

It contains two bioactive isomers called magnolol and honokiol. As for the cardiovascular protection effects, the herbs through magnolol suppress the platelet activation in aggregation step via inhibition of intracellular calcium mobilization and thromboxane formation [15, 37]. Study on inhibition potential of honokiol and magnolol against collagen (10 *µ*g/ml) showed that honokiol is 5 to 10 times more potent than magnolol at even very low concentration and it is concentration dependent. Honokiol and magnolol possess 80% and 55% inhibition on collagen-induced platelet aggregation at the highest concentration. Further test on treated platelets with magnolol and honokiol showed that both had reversible action from collagen-induced platelet aggregation. After conducting the centrifugation of platelet suspension, it is found that there is no presence of lactate dehydrogenase which confirms that both compounds did not cause breakdown of platelets [15].

### 3.5. *Cudrania tricuspidata* Bureau

This plant is also known as* Maclura tricuspidata *Bureau. It is a plant from the Moraceae family. It is a tree native to East Asia and the root has been used as a traditional Chinese medicine for treating dysmenorrhea, rheumatism, and jaundice. The wealthy source of flavonoids and xanthones enhances this plant to be explored. It has been reported to possess antioxidant [38], anti-inflammatory, anticancer [39], antihepatoprotective [40], and anticoagulant properties.

The bioactive compound used, which exhibits the antiplatelet properties obtained from the roots of* Cudrania tricuspidata*, is called cudratricusxanthone A (CTXA). Studies done using mouse platelet showed that CTXA significantly prevented the platelet aggregation induced by thrombin (3 U/ml) in a concentration-dependent manner. At concentration of 10 *µ*M, platelet aggregation reduced to 35% compared to control (80%). CTXA inhibited thrombin-catalyzed fibrin polymerization resulting in a reduction of plasminogen activator inhibitor type 1 (PAI-1) and platelet aggregation [16].

### 3.6. *Angelica sinensis*

*Angelica sinensis* is a type of herb from the family Apiaceae that grows in mountains of China, Korea, and Japan.* Angelica sinensis* is a Chinese traditional herb used for treating dysmenorrhea and it is also used as a food supplement in Korea. Radix* Angelica sinensis* is also officially listed in the Chinese pharmacopoeia. The health benefits are well documented [41]. Conventionally, this plant had been used in the treatment and prevention of thrombosis.

An arterial thrombus is mainly composed of platelet aggregation; hence, the platelets play a vital role in both initiation and growth of thrombi. An initial study has examined the effect of herbs extract named Z-ligustilide, characterized by 3-n-alkylphthalide constituents on atrial-type thrombus formation on a rat arteriovenous shunt model. After administration of the extract (40 mg/kg) for 3 consecutive days orally, thrombus formation was significantly reduced. Further tests on the treated rats showed that the maximum platelet aggregation by ADP (5 *µ*M) had decreased and the result was 3 times greater than aspirin at a dose of 40 mg/kg [17]. Another study done on rabbit showed the same inhibition of ADP-induced platelet aggregation at 10 *µ*M and it was comparable to aspirin at the same dosage. The possible mechanism of action is due to the inhibition of thromboxane A2 formation or Ca^2+^ activation in platelet [18].

### 3.7. *Zingiber officinale* (Ginger)

*Zingiber officinale* from Zingiberaceae family is a medicinal herb that is used in our daily food especially in Asian countries. The benefit of this herb is well known for its antiulcer [42] and antibacterial properties [43]. The common name for this plant is ginger, a traditional medicine with a carminative effect.

This herb has been investigated for its antiplatelet properties. An initial study done on aqueous extract of ginger had shown that it inhibits platelet aggregation induced by AA, adenosine diphosphate, and collagen [19]. A research done on a compound of [8]-gingerol showed that selectively inhibited AA-induced platelet aggregation in washed platelet preparation started at 1 mM up to 20 mM AA concentration. It gave partial inhibition against collagen-induced platelet aggregation at 10 *µ*g/ml [20, 21]. Further studies on [6]- and [8]-gingerol have shown inhibition activity towards COX-1 in human platelet at 0.75 mM AA concentration and using rat basophilic leukemia (RBL-2H3) cells, gingerols at IC_max_ approximately 20 to 25 *µ*M inhibit COX activity by measuring the product of AA metabolism by COX, prostaglandin D2 [3]. These suggest the inhibition mechanism of gingerols possibly due to effect on AA-induced platelet activation. Besides that, another phenolic compound, [8]-paradol, a natural constituent of ginger, also has been found to be the most potent inhibitor against AA-induced platelet aggregation at very low concentration (2 *µ*M). Further test on inhibitory activity of this compound against COX-1 showed the strongest inhibitory activity with IC_50_ values of 4 *µ*M [22]. Overall studies exhibited that ginger mechanism of platelet inhibition through cyclooxygenase-1/thromboxane synthase activity compared to aspirin activity.

### 3.8. *Allium sativum*

*Allium sativum* is also known as garlic. This medicinal herb had been narratively used by veterinarians to treat peripheral venous thrombosis in horses in the French literature a long time ago.

Study on cooked blanched garlic leaves juices on rabbit platelets aggregation induced by ADP and collagen showed significant inhibition with maximum ratio of 87.37% and 86.24%, respectively,* in vitro* and* in vivo*. The inhibition of aggregation pathway mainly blocks the combination of fibrinogen with *µ*Fib-R, which finally results in the inhibition of platelet aggregation proved by the amount of platelet fibrinogen binding detected by flow cytometry [23]. Besides, study on the effect of methanol extract of garlic bulbs (EOG) and three pure components isolated from it (F1, F2, and F3) on human platelet aggregation induced by ADP (10 *µ*M), epinephrine (10 *µ*g/ml), collagen (2 *µ*g/ml), thrombin (0.1 U/ml), arachidonate (1.1 mM), and PAF (10 *µ*M) was conducted. The incubation of platelet rich plasma with EOG inhibits 100% platelet aggregation induced by all of the abovementioned agonists at 68 ± 5 g/ml, 48 ± 2 g/ml, 52 ± 7 g/ml, 95 ± 7 g/ml, 55 ± 3 g/ml, and 60 ± 3 g/ml concentration, respectively. However, for 3 other fractions isolated from it, F3 was shown to have about four times more potency than others using listed agonists at 120 ± 9 g/ml, 60 ± 8 g/ml, 75 ± 11 g/ml, 100 ± 12 g/ml, 30 ± 1 g/ml, and 65 ± 7 g/ml and is also able to induce ADP-rapid deaggregation when added to aggregated platelet. This can indicate the possible mechanism of antiplatelet maybe on the plasma membrane properties. Thrombin-induced release of ATP from platelet also was inhibited by 75–80% after F3 treatment [24].

### 3.9. *Scutellaria baicalensis* Georgi

It is one of the important herbs used in traditional Chinese medicine for treatment of febrile, cough, and liver problem. Its roots have been used for their anti-inflammatory and anticancer effects, for treating bacterial and viral infections, and for reducing the total cholesterol level and hypertension [44]. Several bioactive compounds have been isolated from the root of the plant, namely, baicalein, baicalin, wogonin, and oroxylin A. Baicalin possesses anti-HIV, antitumor, antioxidant, and free radical scavenging effects. Wogonin has antirespiratory syncytial virus, antihepatitis B virus, antitumor, antioxidant, and free radical scavenging effects while oroxylin A has antirespiratory syncytial virus activity [45]. Baicalin had been used in treatment of bronchitis, asthma, and skin allergy [25]. Studies have shown that baicalin inhibited thrombin-catalyzed fibrin polymerization and platelet functions, prolonged aPTT and PT significantly, and inhibited the activities and production of thrombin and FXa.

Further study done on baicalin showed that it suggestively inhibited thrombin- (3 U/ml) and collagen- (1 *µ*g/ml) induced mouse platelet aggregation in a concentration-dependent manner with IC_50_ of baicalin in PRP being 45.6 and 41.1 *µ*M, respectively [25]. A study also demonstrated that baicalin enhanced the phenylephrine-induced contraction through inhibiting the production or/and release of endothelial nitric oxide in the isolated rat mesenteric artery rings [46]. A treatment with active metabolite wogonin named flavonoid wogonoside (WGN) has been shown to inhibit thrombin-catalyzed fibrin polymerization and thrombin-induced platelet aggregation. The inhibition is concentration dependent and at 30 *µ*M of WGN, it is about 50% inhibition with final concentration of thrombin at 3 U/ml. WGN was also shown to elicit anticoagulant effects in mice [26]. Further studies on the root extract of* Scutellaria baicalensis* Georgi have shown a significant inhibition activity of platelet aggregation ratio of rabbits induced by ADP (5 *µ*M) with average inhibition rate of 45.52% at 4.023 mg/ml of extract and that of aspirin group was 54.96% [27].

### 3.10. *Gardenia jasminoides* Ellis

This plant had been known as Zhi Zi in Chinese herb directory. It has been traditionally used as an analgesic for several medical conditions such as pain due to liver problem, dysentery, and trauma for external application [47].

*Gardenia jasminoides* Ellis with its bioactive compounds, iridoid glycosides (IG), has been studied. IGs have been shown to have antithrombotic properties through the inhibition of platelet aggregation with a little effect on the coagulation time of peripheral blood. A study showed that IGs may significantly and dose-dependently reduce arterial thrombus load in a model of carotid artery thrombosis and inhibit collagen-induced platelet aggregation in rats at 1 *µ*g/ml. Pretreatment with IGs for 3 days with dose of 100 mg/kg gave 13% platelet aggregation ratio comparable to aspirin (18%) at 40 mg/kg. [28]. Another compound of* Gardenia jasminoides* Ellis called genipin (GE) with different concentrations has shown significant inhibition on the collagen-induced platelet aggregation* in vitro*, but only a little effect on that induced by ADP and AA [29]. In another* in vivo* thrombosis model, it has also been confirmed that* Gardenia jasminoides* Ellis extract can reduce the weight or length of thrombus. Its mechanism of antiplatelet aggregation and inhibition on thromboembolism was probably related to the activation via inhibiting endothelin-1 (ET-1) expression as a potent vasoconstrictive peptide. GE was found to be very effective in reducing the nuclear factor kappa B (NF-*κ*B) by disturbing the I*κ*B degradation. NF-*κ*B is an inducer of cyclooxygenase-2 (COX-2) expression. By reducing NF-*κ*B activity, the production of COX-2 and inducible nitric oxide synthase (iNOS) is downregulated [30, 48].

## 4. Conclusion

This review focused on the availability of medicinal herbs that possess antiplatelet properties. Most studies are still at the preliminary stage as many steps are required so that the herbs can be used as a substitution or can be combined with current antiplatelet agents such as aspirin and clopidogrel.

## Figures and Tables

**Table 1:** Summary of inhibition of platelet activation by medicinal herbs.

Reference	Plants	Compound	Dose of plants' extract	Agonist and dosage	Postulated antiplatelet properties	*In vivo* or *in vitro*
[6–8]	Turmeric (*Curcuma longa*)	Ar-turmerone, curcumin	100 *µ*g/ml, 500 *µ*M	Collagen (2 *µ*g/ml), ADP (10 *µ*M), and thrombin (0.64 U/ml)	Adhesion	*In vivo*

[12, 13]	Barberry (*Berberis vulgaris*)	Berberine	30 mg/kg	Collagen	Adhesion	*In vivo*

[14]	*Osmanthus fragrans*		2.8 mg/ml	Collagen (1 mg/ml) and ADP (5 mM)	Adhesion, secretion	*In vitro*

[15]	Magnolia (*Magnolia officinalis*)	Magnolol, honokiol		Collagen (10 *µ*g/ml)	Adhesion	*In vitro*

[16]	*Cudrania tricuspidata *Bureau	Cudratricusxanthone A	10 *µ*M	Thrombin (3 U/ml)	Aggregation	*In vitro*

[17, 18]	Dong quai (*Angelica sinensis)*	Z-Ligustilide	40 mg/kg	ADP (5 *µ*M)	Secretion	*In vivo*

[19–22]	Ginger (*Zingiber officinale*)	Gingerol, paradol	25 *µ*M	AA (20 mM) and AA (2 *µ*M)	Secretion	*In vitro*

[23, 24]	Garlic (*Allium sativum*)	Methanol extract	48–95 g/kg	ADP (10 *µ*M), epinephrine (10 *µ*g/ml), collagen (2 *µ*g/ml), thrombin (0.1 U/ml), AA (1.1 mM), and PAF (10 *µ*M)	Adhesion, secretion, and aggregation	*In vitro*, *in vivo*

[25–27]	*Scutellaria baicalensis* Georgi	Baicalin, wogonoside	45.6 *µ*M, 30 *µ*M	Thrombin (3 U/ml), collagen (1 *µ*g/ml), and ADP (5 *µ*M)	Adhesion, secretion	*In vitro*

[28–30]	*Fructus gardenia*	Iridoid glycosides, genipin	100 mg/k	Collagen (1 *µ*g/ml)	Adhesion, vasoconstriction	*In vivo*
